# Supplementary material for: Quantifying thermal adaptation of soil microbial respiration
Source: Nat Commun. 2023 Sep 6;14:5459. doi: 10.1038/s41467-023-41096-x (PMC10482979; doi:10.1038/s41467-023-41096-x)
Supplement: Supplementary file 1 — Supplementary Information [file 41467_2023_41096_MOESM1_ESM.pdf]

Supplementary Figures

Quantifying thermal adaptation of soil microbial respiration

Charlotte J. Alster, Allycia van de Laar, Jordan P. Goodrich, Vickery L. Arcus, Julie R. Deslippe, Alexis J. Marshall, Louis A. Schipper

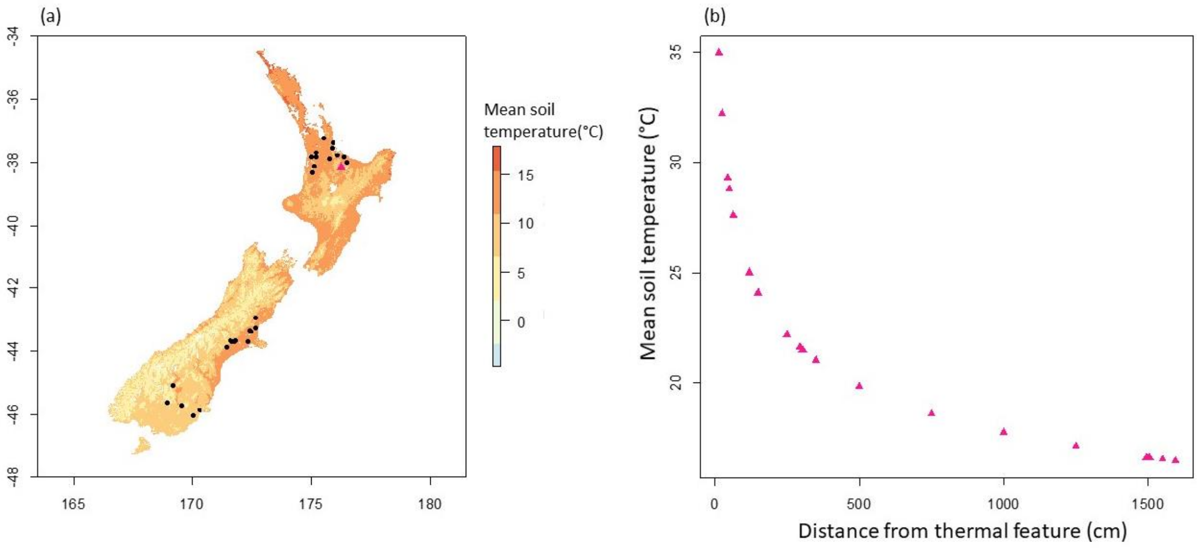

Fig. S1. (a) Map of New Zealand coloured with mean soil temperature. (b) Plot of mean soil temperature versus distance from the geothermal feature. In both panels, each point represents a different sampling location with pink triangle(s) indicating soil samples from the geothermal gradient. Soil temperatures represent ~5 cm depth.

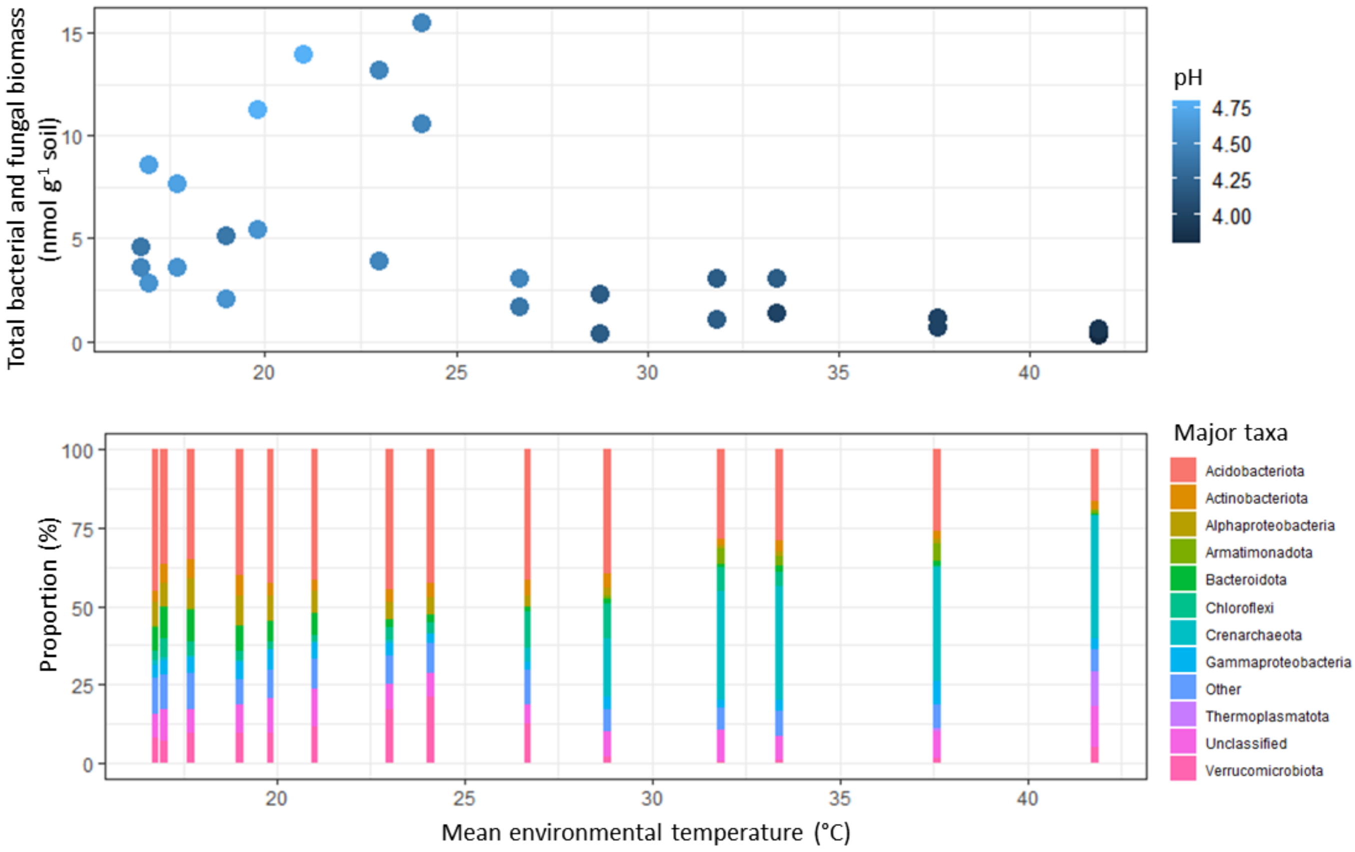

Fig. S2. (a) Total bacterial and fungal biomass and (b) relative abundance of each major prokaryotic taxa found along the geothermal gradient. In panel (b), proportions are averaged between the two replicates at the same temperature when applicable.

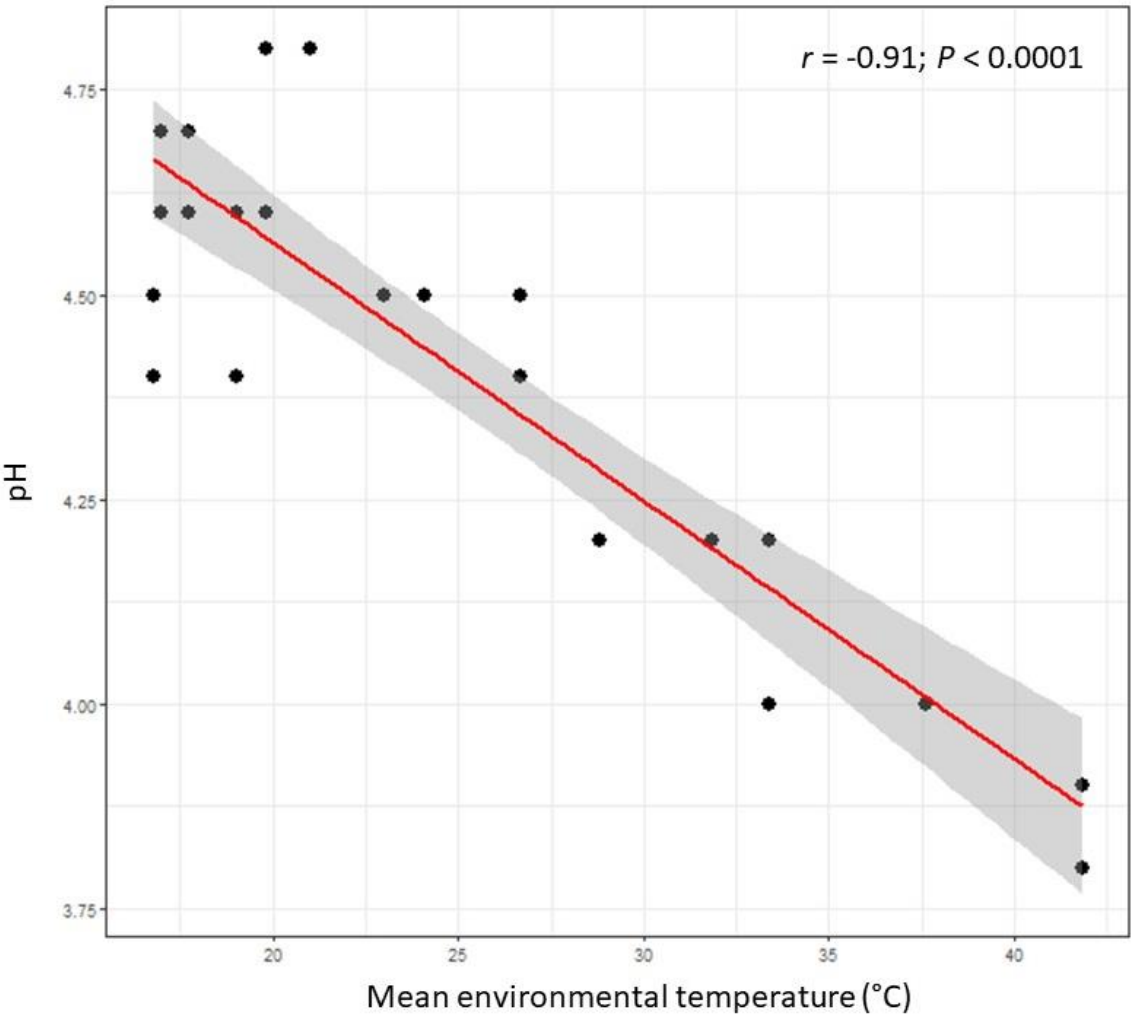

Fig. S3. Relationship between pH and mean environmental temperature along the geothermal gradient. The red line indicates best fit with the grey shading corresponding to the 95% confidence interval.

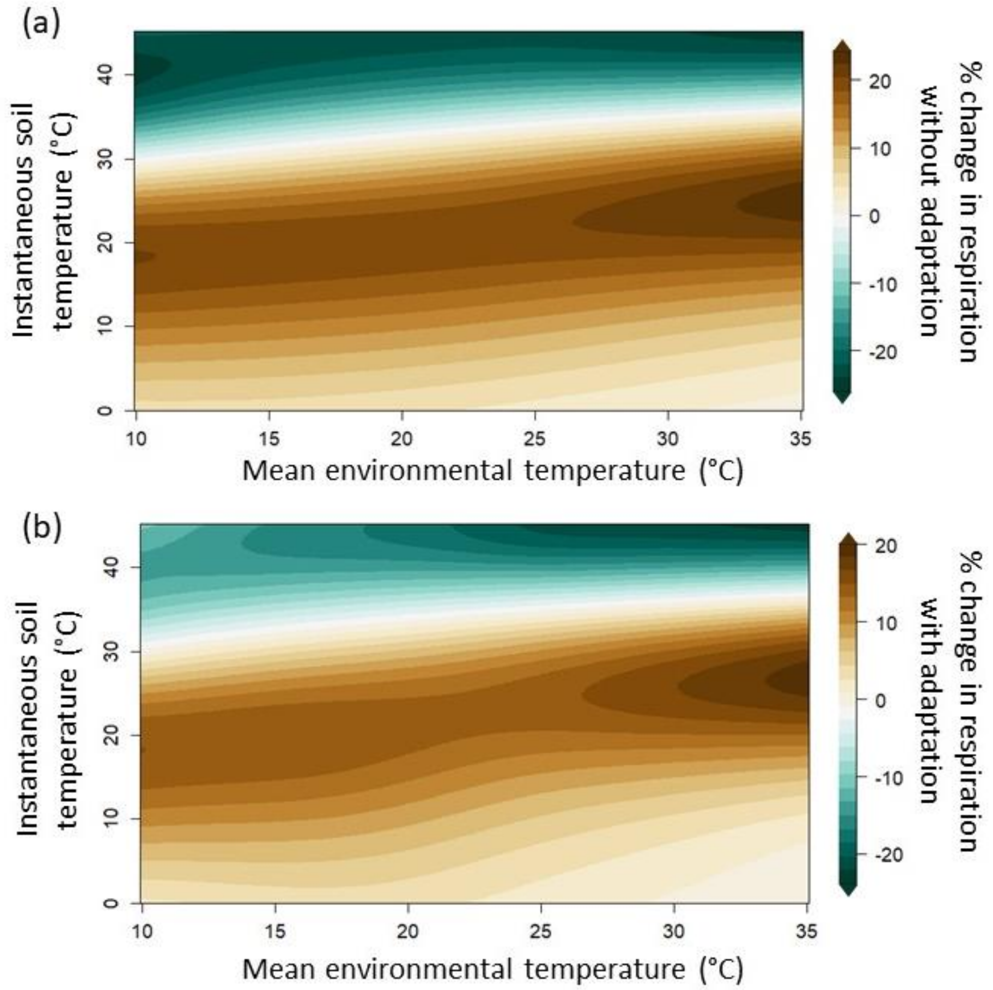

Fig. S4. (a) Percent change in the total respiration rate with 4.5°C of soil warming assuming no thermal adaptation of the microbial community. (b) Percent change in the total respiration rate with 4.5°C of warming with thermal adaptation of the microbial community.

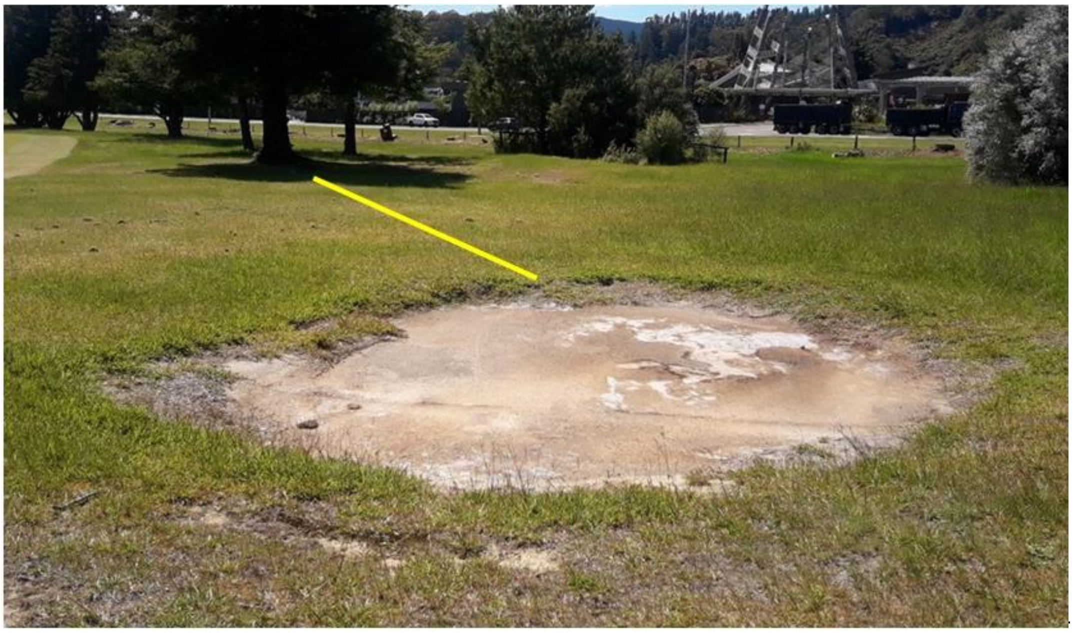

Fig. S5. Photograph of the heated ground feature. Soil samples were collected across 1 m transects perpendicular to the yellow line (~16 m) as soil temperatures decreased exponentially moving away from the thermal feature (Fig. S1b).

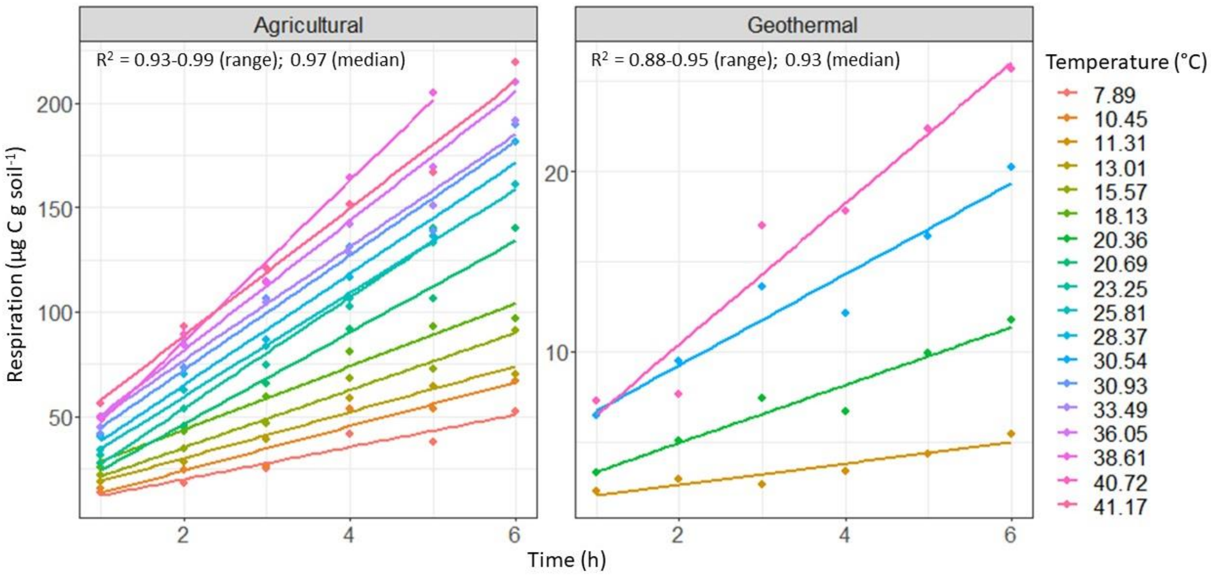

Fig. S6. Respiration measured over time for an agricultural and geothermal soil. Colour indicates incubation temperature and lines indicate best fit. Data is presented only for temperatures below 42°C since the MMRT model fits were restricted to that temperature range.

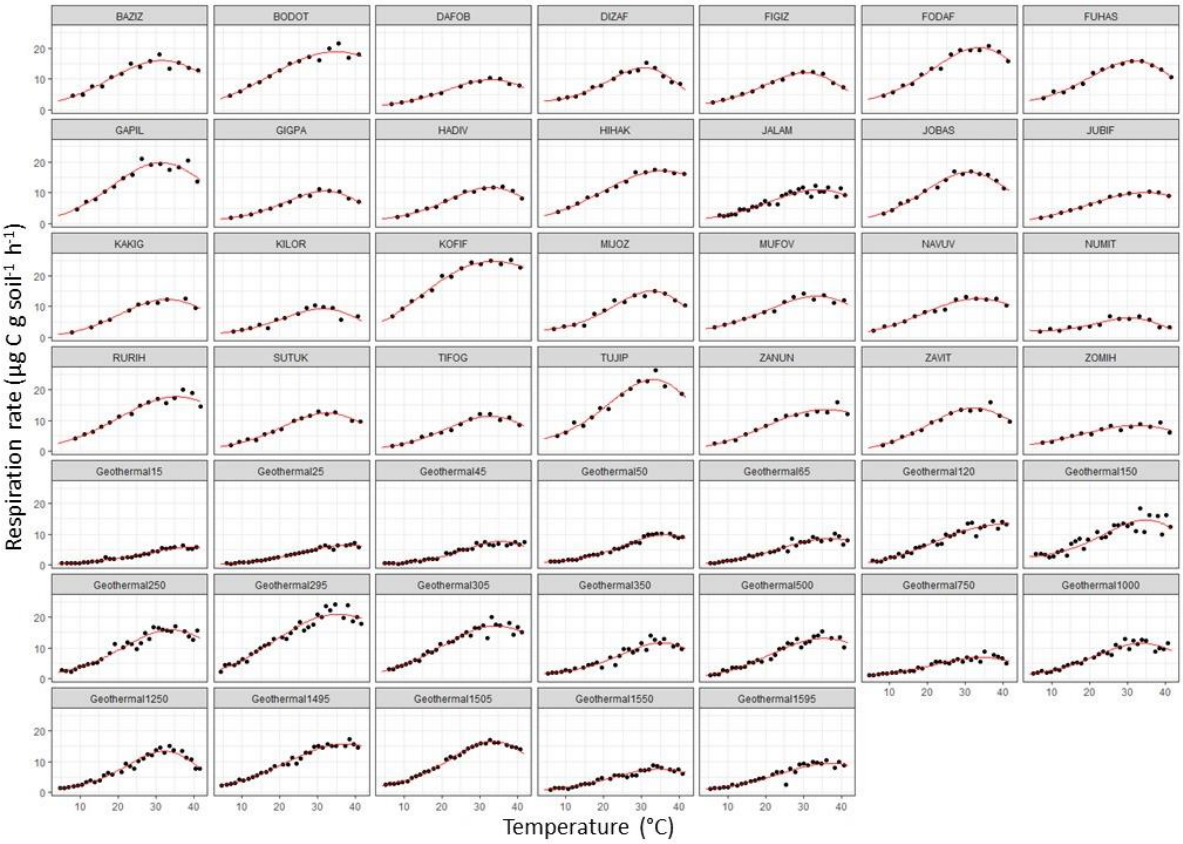

Fig. S7. Temperature response of soil microbial respiration with each panel representing a different soil sample. The black points represent individual respiration rate measurements at each discrete incubation temperature and the red lines indicate the modified MMRT model fits.
