# Supplementary material for: Quantifying thermal adaptation of soil microbial respiration
Source: Nat Commun. 2023 Sep 6;14:5459. doi: 10.1038/s41467-023-41096-x (PMC10482979; doi:10.1038/s41467-023-41096-x)
Supplement: Supplementary file 3 — Description of Additional Supplementary Files [file 41467_2023_41096_MOESM3_ESM.pdf]

Description of Additional Supplementary Files

Supplementary Data Tab 1: Site characteristics. Site and experimental details for each of the sampled soils.

Supplementary Data Tab 2: ICPMS data. Elemental data from along the geothermal gradient. All data is presented as ppm.

Supplementary Data Tab 3: MET and pH regression. AIC and R^2 values for each regression model. Note, the column entitled "All data" uses spatial autoregression models, while the "geothermal gradient only" columns use linear regression. Because an adjusted R^2 cannot be calculated for the spatial autoregression models, a Nagelkerke pseudo-R^2 is reported instead.

Supplementary Data Tab 4: ASV counts. ASV counts from along the geothermal gradient. Column headers indicate distance (cm) from the geothermal feature. Sinlgetons are removed.

Supplementary Data Tab 5: Taxonomy. Taxonomic information for the samples collected along the geothermal gradient.

Supplementary Data Tab 6: Fig. 4 values. Differences in percent potential respiration between the adapted and unadapted temperature response curves at 4.5°C of warming.

Supplementary Data Tab 7: CO2 flux values. Raw data of the respiration rates (Rs) at each measured incubation temperature for each sampled soil.

Supplementary Data Tab 8: AIC values MMRT. AICc values for MMRT and the modified version of MMRT model fits for each of the temperature response curves.

Supplementary Data Tab 9: MMRT values. Parameter values estimated from the modified MMRT model fits for each temperature response curve.

Supplementary Data Tab 10: Fatty acid biomarker. Fatty acid biomarker designation.
