# Supplementary material for: Quantifying thermal adaptation of soil microbial respiration
Source: Nat Commun. 2023 Sep 6;14:5459. doi: 10.1038/s41467-023-41096-x (PMC10482979; doi:10.1038/s41467-023-41096-x)
Supplement: Supplementary file 5 — Reporting Summary [file 41467_2023_41096_MOESM5_ESM.pdf]

nature portfolio

Corresponding author(s): Charlotte Alster

Last updated by author(s): Aug 16, 2023

Reporting Summary

Nature Portfolio wishes to improve the reproducibility of the work that we publish. This form provides structure for consistency and transparency in reporting. For further information on Nature Portfolio policies, see our Editorial Policies and the Editorial Policy Checklist.

Statistics

For all statistical analyses, confirm that the following items are present in the figure legend, table legend, main text, or Methods section.

n/a	Confirmed	
☐	☒	The exact sample size (*n*) for each experimental group/condition, given as a discrete number and unit of measurement
☐	☒	A statement on whether measurements were taken from distinct samples or whether the same sample was measured repeatedly
☐	☒	The statistical test(s) used AND whether they are one- or two-sided<br>*Only common tests should be described solely by name; describe more complex techniques in the Methods section.*
☐	☒	A description of all covariates tested
☐	☒	A description of any assumptions or corrections, such as tests of normality and adjustment for multiple comparisons
☐	☒	A full description of the statistical parameters including central tendency (e.g. means) or other basic estimates (e.g. regression coefficient) AND variation (e.g. standard deviation) or associated estimates of uncertainty (e.g. confidence intervals)
☐	☒	For null hypothesis testing, the test statistic (e.g. *F*, *t*, *r*) with confidence intervals, effect sizes, degrees of freedom and *P* value noted<br>*Give P values as exact values whenever suitable.*
☒	☐	For Bayesian analysis, information on the choice of priors and Markov chain Monte Carlo settings
☒	☐	For hierarchical and complex designs, identification of the appropriate level for tests and full reporting of outcomes
☒	☐	Estimates of effect sizes (e.g. Cohen's *d*, Pearson's *r*), indicating how they were calculated

Our web collection on statistics for biologists contains articles on many of the points above.

Software and code

Policy information about availability of computer code

Data collection	Climate monitoring network data from NIWA using 'clifro' package in R<br>Soil characterization information was obtained through S-map
Data analysis	Custom code is available at Zenodo (10.5281/zenodo.8248107)<br>Spatial autoregression model: ‘spatialreg’ package in R<br>Sequencing data processing and analysis: Cutadapt v2.3, DADA2 v1.14.1 in R, ASV assignment with DECIPHER v2.22.0 using the SILVA v138 database, MAFFT v7, FastTree v2.1.11, and the 'phyloseq', 'vegan', and 'picante' packages in R

For manuscripts utilizing custom algorithms or software that are central to the research but not yet described in published literature, software must be made available to editors and reviewers. We strongly encourage code deposition in a community repository (e.g. GitHub). See the Nature Portfolio guidelines for submitting code & software for further information.

Data

Policy information about availability of data

All manuscripts must include a data availability statement. This statement should provide the following information, where applicable:

- Accession codes, unique identifiers, or web links for publicly available datasets
- A description of any restrictions on data availability
- For clinical datasets or third party data, please ensure that the statement adheres to our policy

All data is available in the Supplementary Tables, at Figshare (https://figshare.com/s/3746ef75599e608a5984), and/or at https://www.ncbi.nlm.nih.gov/sra/PRJNA1002820 under the BioProject accession number PRJNA1002820. Temperature data from sites not along the geothermal gradient was sourced from NIWA climate monitoring network data (https://cliflo.niwa.co.nz/).

Human research participants

Policy information about studies involving human research participants and Sex and Gender in Research.

Reporting on sex and gender	N/A
Population characteristics	N/A
Recruitment	N/A
Ethics oversight	N/A

Note that full information on the approval of the study protocol must also be provided in the manuscript.

Field-specific reporting

Please select the one below that is the best fit for your research. If you are not sure, read the appropriate sections before making your selection.

☐ Life sciences ☐ Behavioural & social sciences ☒ Ecological, evolutionary & environmental sciences

For a reference copy of the document with all sections, see nature.com/documents/nr-reporting-summary-flat.pdf

Ecological, evolutionary & environmental sciences study design

All studies must disclose on these points even when the disclosure is negative.

Study description	We sampled soils (n=48) that varied in mean annual temperature (11-35°C) and created 48 unique temperature response curves of soil microbial respiration under substrate saturating conditions in the laboratory by incubating theses soils at 11+ temperatures (~4-42°C). After estimating the Topt and Tinf from each curve, we then fit two spatial simultaneous autoregresion models, to account for location effect, with Topt and Tinf as the dependent variables and mean environmental soil temperature as the independent variable.
Research sample	We measured CO2 flux from soils with and without added glucose at 11+ distinct temperatures from 48 different samples to create temperature response curves. Each temperature response curve was fit with a modified MMRT model to use for the analysis and interpretation. Other metadata was also collected alongside each soil sample, such as pH and soil moisture. For the soil samples from the geothermal gradient, additional data was collected to characterize the microbial community and chemical composition (n=28).
Sampling strategy	Soil was sampled from the top 7.5 cm. Several soil cores from each location were mixed to homogenize and provide sufficient soil for the laboratory incubations and/or other analyses. Our analyses revealed very high confidence in our regression (see Fig. 2) and indicated sufficient sample size. Additionally, the large number of total data points from all of the temperature response curves combined corresponded to high enough detail for smoothing of the scaled curves over the 10-35°C temperature range.
Data collection	Soil samples were collected by the research team at Manaaki Whenua - Landcare Research or by A.v.d.L. Samples were either refrigerated at 10°C until the incubations could occur or placed at room temperature if incubations would be occurring within one week of collection. CO2 flux data from the incubations was measured by A.v.d.L. The DNA extraction and amplification was done by C.A. and the PLFA measurements were done by J.D. Soil collected for the sequencing and biomass measurements were placed on ice in the field. The samples for PLFA were shipped on ice overnight and then lyophilized. The samples for DNA analysis were stored at -20C until processing.
Timing and spatial scale	Soil samples were collected between 30 November 2020 and 3 December 2021 with laboratory incubations of those soils conducted between 1 December 2020 and 20 December 2021. Sampling and incubation dates are reported in Table S1. Soils for DNA sequencing were collected on 29 March 2021 and soils for PLFA were collected on 31 May 2021. We collected samples from a diverse range of mean annual soil temperatures.

Data exclusions	One site was excluded from the final analysis since the MMRT model would not converge (final n=47). Site details and the raw CO2 flux data for this sample are included in Tables S1 and S7. Seven samples were also excluded from the microbial community analysis since they had low sequence reads (<1200) (final n=21). These details are reported in the Methods section.
Reproducibility	Temperature response curves included at least 11 distinct temperatures. Soils were sampled from 48 locations for the regression analysis. Microbial community analyses and biomass were duplicated twice at 14 locations along the geothermal gradient, however not all replication attempts were successful (see Methods for details).
Randomization	Sites for soil sampling from around NZ were selected randomly while maximizing geographic distance (see Methods for more details). However, due the length of nation-wide soil sampling campaign and intensity of the incubation measurements we conducted, not all of the samples were included in this experiment. Once a sufficient sample size had been obtained for our analysis, as determined by a post-hoc power analysis (power > 0.99), we ceased data collection for this experiment. Soil sampling occurred in a non-random order due to logistical constraints, but incubation experiments were conducted in a random order once they arrived in the lab. Samples from the geothermal gradient were selected randomly within assigned temperature zones and were also incubated in a random order in the lab. Despite sampling on different days, we did not find a significant relationship between sampling date and the temperature response (P > 0.05). Samples designated for microbial community analysis were also processed in a random order. We used the randomization function in Excel to designate in-lab process order.
Blinding	Blinding was not used in this study.

Did the study involve field work? ☒ Yes ☐ No

Field work, collection and transport

Field conditions	Site characteristics for each soil sample (including mean annual soil temperature, soil classification, land use, C/N, moisture, pH, and sampling date) are reported in Table S1.
Location	To protect landowner privacy, exact coordinates are not provided. However, approximate locations of each sample are shown in Fig. S1.
Access & import/export	All samples were taken from within New Zealand, so no import/export permits were needed. For samples taken at the Arikikapakapa golf course, permission for sampling was obtained from the groundskeeping team. For the remaining sites, Manaaki Whenua - Landcare Research obtained permission from each landowner prior to soil sampling.
Disturbance	Disturbance was minimal and was restricted to soil removed as 2.5 cm cores or small pits. When applicable, soil was refilled into those the holes.

Reporting for specific materials, systems and methods

We require information from authors about some types of materials, experimental systems and methods used in many studies. Here, indicate whether each material, system or method listed is relevant to your study. If you are not sure if a list item applies to your research, read the appropriate section before selecting a response.

Materials & experimental systems

n/a	Involved in the study	
☒	☐	Antibodies
☒	☐	Eukaryotic cell lines
☒	☐	Palaeontology and archaeology
☒	☐	Animals and other organisms
☒	☐	Clinical data
☒	☐	Dual use research of concern

Methods

n/a	Involved in the study	
☒	☐	ChIP-seq
☒	☐	Flow cytometry
☒	☐	MRI-based neuroimaging
